# Case report: Evolution of pulmonary manifestations and virological markers in critical COVID-19 infection in Bruton’s agammaglobulinemia

**DOI:** 10.3389/fimmu.2022.1057065

**Published:** 2022-11-24

**Authors:** Nina Rise, Toke Touborg, Ditte Helene Lundsted, Michael Dalager-Pedersen, Trine H. Mogensen

**Affiliations:** ^1^ Department of Infectious Diseases, Aalborg University hospital, Aalborg, Denmark; ^2^ Department of Biomedicine, Aarhus University, Aarhus, Denmark; ^3^ Department of Clinical Microbiology, Aalborg University Hospital, Aalborg, Denmark; ^4^ Department of Infectious Diseases, Aarhus University Hospital, Aarhus, Denmark

**Keywords:** Bruton’s agammaglobulinemia, COVID-19, SARS-CoV-2, inborn error of immunity, viral Evolution, vaccination

## Abstract

Despite several reports and small case series on the disease course of SARS-CoV-2 infection in patients with inborn errors of immunity (IEI), including X-linked agammaglobulinemia (XLA), this topic remains incompletely described. Here we present the case of a 38-year-old unvaccinated man with XLA, who acquired SARS-CoV-2 infection and experienced a protracted disease course with 47 days of SARS-CoV-2 positivity, critical COVID-19 with respiratory insufficiency necessitating intensive care and ventilatory support, and prompting repeated intensified treatments with remdesivir, dexamethasone, and monoclonal antibodies to eventually control infection. We describe the disease course and treatment and review the current literature on COVID-19 susceptibility and evidence for vaccine efficacy in patients with XLA.

## Introduction

Bruton’s agammaglobulinemia is a rare X-linked genetic disorder first described by Ogden Bruton in 1952 (1). This condition is caused by mutations in the gene encoding Bruton’s tyrosine kinase (BTK) (2), which is a cytoplasmatic tyrosine kinase pivotal to B-cell development, differentiation and signalling (3). Patients with inherited compromised BTK function present with a classical X-linked agammaglobulinemia (XLA) phenotype at around 4 to 6 months of age, when maternally transmitted IgG levels have declined. These patients suffer from severe panhypogammaglobulinemia and thus have a significantly compromised humoral response to infections, causing increased susceptibility to often life-threatening recurrent sinopulmonary infections, viral gastrointestinal infections and chronic meningoencephalitis following enterovirus infection (2). Multiple recurrent respiratory infections and the accompanying inflammation leads to bronchiectasis, thereby maintaining a vicious circle of inflammation, tissue destruction, and further susceptibility to pulmonary infections. Bronchiectasis and respiratory complications are leading causes of mortality and morbidity in patients with XLA, who had a significantly reduced mean lifetime expectancy before the advent of immunoglobulin substitution therapy. However, with these modern therapies and high standardized healthcare systems patients with XLA are now expected to live well into adulthood (2).

Current treatment of XLA is lifelong immunoglobulin replacement therapy (IgRT) and prophylactic antibiotics. IgRT is administered either subcutaneously or intravenously, with a therapeutic aim of IgG serum levels of more than 5 g/L and preferably 8 g/L or higher (4). IgRT products however lack IgA and IgM due to safety and efficacy concerns, which may contribute to an increased tendency to gastro- and sinopulmonary infections, despite normal IgG levels during IgRT treatment (2, 5). Substitution of BTK functions besides that of B-cell development remains therapeutically unaddressed. B-lymphocytes exert a plethora of functions in the immune system and their response to infection is crucial in immune defences and homeostasis. Secretion of antibodies and immunomodulatory molecules (such as interferon (IFN)-γ, interleukin (IL)-6, and IL-10), antigen presentation, regulation of T-lymphocytes and dendritic cells, are all examples of immune processes with critical B-lymphocyte involvement.

## Results

### Case report

A 38-year-old male patient with known XLA presented to hospital after 19 days (D19) of symptomatic COVID-19. He was diagnosed with XLA in early childhood with a pathogenic mutation in the *BTK* gene (BTK c.215_216insA [NM_000061.2]. He had received immunoglobulin substitution therapy since XLA diagnosis at the age of eight months, most recently by subcutaneous HyQvia 300 mL (30 g) every 4 weeks. As treatment compliance had been relatively poor, he had suffered from numerous pneumonias with *Streptococcus pneumonia* and *Haemophilus influenzae* over the years. Most recent immunoglobulin G (IgG) values were around 6-7 g/L and thus below the recommendations of IgG > 8 g/L in patients with XLA, and with IgA and IgM levels below detection limit in accordance with the diagnosis. Moreover, he was smoking despite counselling against it. His latest x-ray of the lungs prior to COVID-19 was described with minor atelectasis and bronchiectasis in the medial lobe and bullous emphysema bilaterally. No pulmonary function test or chest CT had been performed over many years due to poor compliance. As a consequence of insufficient control of infection, he had received prophylactic antibiotics (Sulfamethoxazole/Trimethoprim 400/80 mg daily) over the past three years. He had been offered the COVID-19 mRNA vaccine (Pfizer-BioNTech), which he had declined due to fear of adverse effects, despite much encouragement to receive the vaccine.

### Disease course

**Figure 1:**
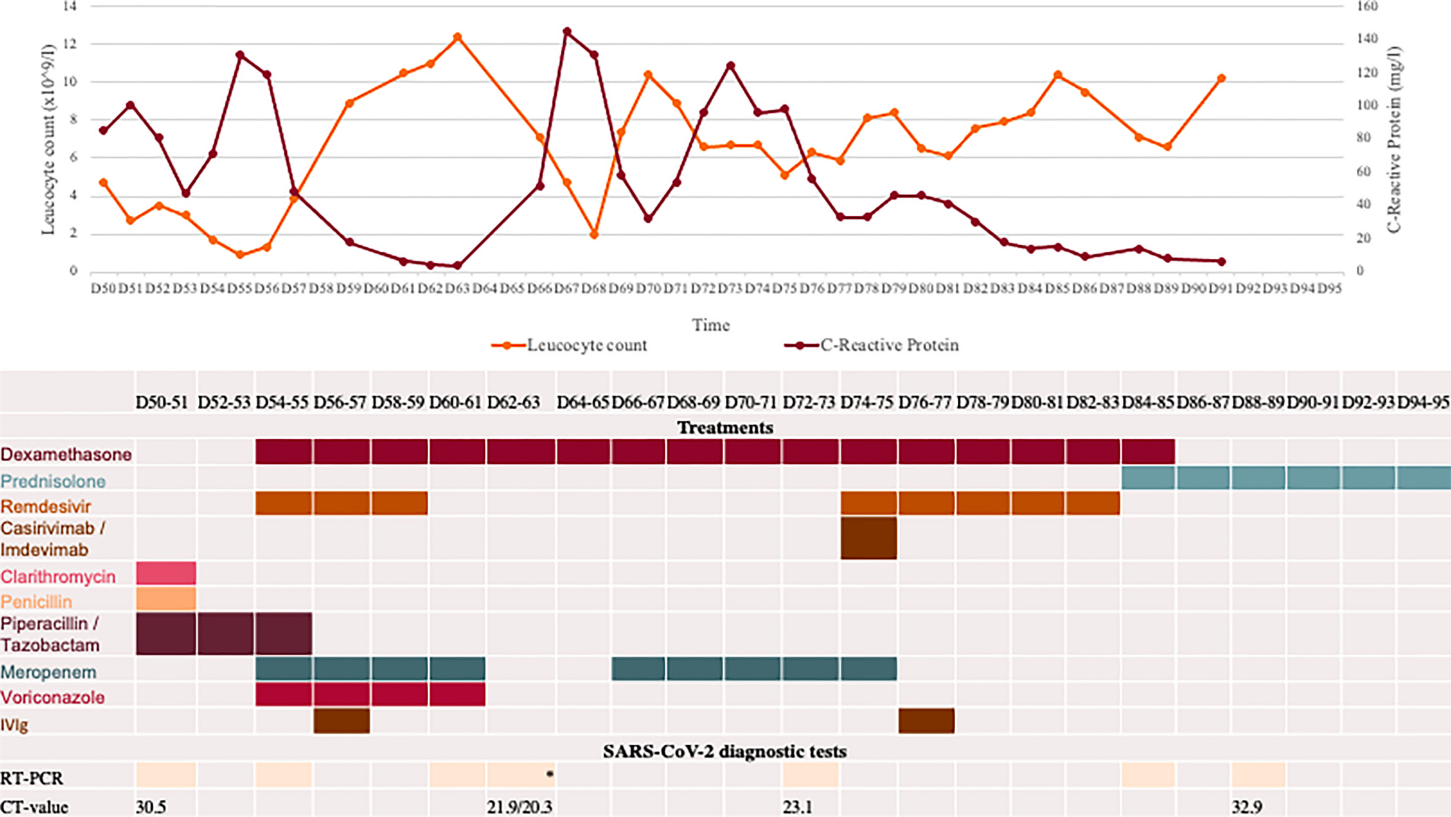
Overview of blood test, COVID-19 diagnostic analyses, and medications during second hospital admission starting at day 50 (D50) after first positive SARS-CoV-2 test. All RT-PCR tests were positive for SARS-CoV-2 B.1.617.2 (Delta variant) performed on material from nasopharyngeal swab, except *two tests performed on BAL material. PIT: piperacillin/tazobactam. Cut-off for positive SARS-CoV-2 PCR is a Ct value of 38. Pip/Tazo, piperacillin/tazobactam; IVIG, intravenous immunoglobulin; Casirivi/Imdevi, casirivimab-Imdevimab.

On day 0, D0, the patient started to experience symptoms of mild upper respiratory tract infection. He sought a diagnostic test for COVID-19 on D5 and his reverse transcription polymerase chain reaction (RT-PCR) nasal swab came out positive for SARS-CoV-2, B.1.617.2 (Delta variant variant). Due to persisting symptoms, he was admitted to hospital on D20. On admission, the patient presented with fever (39.0°C) but was not hypoxic, and chest x-ray showed no signs of pneumonia. On suspicion of COVID-19 with bacterial superinfection intravenous antibiotics (piperacillin/tazobactam) were administered along with oral dexamethasone. Remdesivir was initially omitted due to the prolonged duration of symptoms. Instead, the patient was treated with the anti-SARS-CoV-2 monoclonal antibody (mAb) Sotrovimab on D21. After two days in hospital, he was discharged to complete a 10-day course of dexamethasone. However, his respiratory symptoms gradually progressed with severe dyspnoea, cough, and high fever (40.3°C), leading to re-admission one month later at D50 (**Figure 1**). RT-PCR performed upon admission was positive for SARS-CoV-2 B.1.617.2 (Delta variant, cycle threshold (Ct)-value of 30.5). Computed tomography (CT) scan of the chest showed diffuse bilateral pulmonary infiltrates. No restriction perfusion Due to the long timespan since initial COVID-19 diagnosis, he was initially suspected to be suffering from bacterial pneumonia rather than COVID-19. Hence, intravenous piperacillin/tazobactam and oxygen (3 L/min) was initiated with no concomitant treatment for COVID-19 (**Figure 1**).

**Figure 2:**
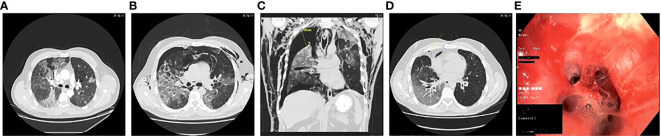
Computed tomography scans showing pulmonary infiltrates, pneumothorax and subcutaneous emphysema at **(A)** D50; **(B)** D70; **(C)** D93; **(D)** D153; and **(E)** clinical photo from bronchoalveolar lavage showing mucoid impaction at D63.

In the days following admission, the patient’s condition continued to deteriorate. It was decided to reinstitute COVID-19 treatment with dexamethasone and remdesivir four days after admission, on D54 in combination with intravenous meropenem. Moreover, empirical voriconazole was administered to cover a possible *Aspergillus* spp. co-infection, which was suspected due to the high dose dexamethasone treatment previously reported to increase the risk of infection with *Aspergillus* spp.in critically ill COVID-19 patients in the ICU patients (6). The following day his condition further deteriorated with increased need for oxygen therapy (10 L/min). He received an infusion of intravenous immunoglobulin (IVIG) (Privigen 30 g) and was admitted to the intensive care unit (ICU) for stabilisation. During admission his IgG levels were maintained within the normal range (IgG>6 g/L). Due to increasing alanine aminotransferase levels, remdesivir and voriconazole were discontinued after five days of treatment, while meropenem and dexamethasone treatment continued for six and ten days respectively (**Figure 1**). A positron emission tomography scan performed on D60 to rule out other infection, inflammation/vasculitis or malignancy showed continuous pulmonary bilateral pneumatic infiltrates, and a bronchoalveolar lavage (BAL) was performed on D63. Material from the lungs was PCR positive for SARS-CoV-2 B.1.617.2 (Ct values 20.3 and 21.86), but negative for aspergillosis, *Pneumocystis jirovecii*, *Legionella pneumophila*, *Mycoplasma pneumonia*, and *Chlamydia pneumonia* and with sterile cultures. At this time, the patient was readmitted to the ICU. One week later, D70, the patient experienced respiratory deterioration with increasing demand for oxygen therapy. Chest-CT revealed a pneumothorax of the right lung, pneumomediastinum, and subcutaneous emphysema (**Figure 2**). The latter presented as a visible swelling around the patient’s neck and in a few days progressed to swelling of the face and orbital regions, prompting re-admission for the third time to the ICU. Chest tube insertion was performed, and the patient was placed on high-flow oxygen therapy (20-29 L/min). His condition continued to deteriorate, and plasma-C Reactive Protein (CRP) levels rose. Therefore, it was decided to introduce yet another round of treatment for COVID-19. Remdesivir was readministered along with yet another mAb treatment (Casirivimab-Imdevimab, REGN-COV2, 600 + 600 mg), with potentially increased efficacy against the SARS-CoV-2 variant in the patient. and infusion with immunoglobulin (Privigen 30 g). During the following days, the patient’s condition gradually improved. He spent another 14 days in the ICU before being moved to the ward. On D94 the pneumothorax and subcutaneous emphysema had regressed sufficiently to remove the chest tube and the patient was discharged for follow-up in the outpatient clinic (**Figure 2**). One-and-a-half month after discharge, D138, the patient still suffered some degree of pulmonary sequalae with persistently positive SARS-CoV-2 RT-PCR tests, all showing infection with the Delta variant.

### Paraclinical virological findings

During the disease course a total of 11 respiratory samples were collected and analysed using reverse transcription PCR (RT-PCR) for SARS-CoV-2 (**Figure 1**). SARS-CoV-2 were detected in every sample. Generally, the Ct values increased after D73 from 23.1 to 32.9/34.9 reflecting a decreasing viral load during the last period of hospitalisation, although the precise relation between specific Ct values and viral load may be uncertain and also depends on the technological platform and viral strain (7). CoviDetect™ COVID-19 Mutation RT-PCR Assay (PentaBase) was used to detect the mutation signatures of major circulating variants, later confirmed by whole exome sequencing, and were found to belong to B.1.617.2 (Delta variant). Continuous development of three different viral amino acid substitutions were detected, including E:T30I, ORF1a:G3072C and S:E340V. Among these, mutations at position 340 of the Spike protein has been associated med sotrovimab resistance (8). This was the rationale behind introduction of a second round of mAb treatment with Casirivimab-Imdevimab with presumed increased efficacy against the evolving variants of SARS-CoV-2 in the patient (9).

## Discussion

### Outcome of SARS-CoV-2 infection and viral clearance in patients with XLA

Several risk factors have been identified for developing severe disease from SARS-CoV-2 infection in the general population, including age, male gender, as well as co-morbidities such as cardiovascular and pulmonary disease, obesity, diabetes, and liver or kidney dysfunction. In addition, a number of inborn errors of immunity (IEI) predispose to severe/critical COVID-19 as well as increased mortality from infection, although with an extensive variability depending on the different immune cell subsets and signaling molecules affected (10). Initial descriptions of SARS-CoV-2 infection and COVID-19 in patients with XLA reported mild and asymptomatic cases (10). It was even been suggested that B-cell depletion and compromised BTK function may be protective against the cytokine storm seen in severe and critical COVID-19, reflecting an important role of B cells in COVID-19 pathogenesis (10, 11). This was based on the findings that patients with severe COVID-19 have been shown to display increased BTK activation and elevated IL-6 production (12), together with clinical trials demonstrating increased survival of COVID-19 in patients receiving IL-6-blockade with tocilizumab (13). However, the majority of the accumulating literature indicates an increased risk of severe infection, longer disease duration and poorer outcome in patients with XLA (14–17). In a literature review, Ponsford et al. summarized the outcome of SARS-CoV-2 infection in 28 patients with XLA and found that 22/28 (79%) were admitted to hospital, and of those, 11% were admitted to the ICU (18). Furthermore, the mean time until viral clearance reported in 8/23 cases was 38.3 days compared to 17 and 17.2 days of viral shedding from the upper and lower respiratory tract, respectively (19). Moreover, they found a relatively high case fatality rate of 4%, especially given the small sample size and young age of the patient cohort. In a study by Giardino et al., a hospitalization rate of 20% was recorded in a cohort of 114 patients with IEI. Among the hospitalized patients, 82% suffered from immunodeficiencies within the humoral compartment and had lower B-cell count than the non-hospitalized patient group (20), suggesting antibody deficiency as a major risk factor for severe disease. Moreover, the occurrence of prolonged infection was significantly higher in hospitalized patients than non-hospitalized patients, and the prevalence of prolonged infection in XLA was increased to 40% of patients. Interestingly, viral shedding in patients with humoral deficiencies was longer compared to other IEI (20). Brown et al. observed clinical features of COVID-19 disease similar to the general population, although with prolonged duration of symptoms (median 64 days) and relapsing infections in 31 patients with antibody deficiencies or profoundly reduced peripheral B-cell levels experiencing long lasting (>21 days) or relapsing COVID-19 episodes. These autothors concluded that COVID-19 can present as a chronic or relapsing disease in patients with antibody deficiency, similar to the case described here. Monotherapy with Remdesivir was frequently associated with treatment failure, whereas combination therapy consisting of Remdesivir and an antibody-based therapeutic resulted in viral clearance in 13/14 episodes (92.8%) (21). These findings are supported by other reports of prolonged SARS-CoV-2 infection in other B-cell deplete conditions, including multiple myeloma, or secondary to B cell depletion with anti-CD20 antibodies (Rituximab), resulting in absent neutralizing antibody responses to SARS-CoV-2 (22). Most recently, an overview of SARS-CoV-2 infection in patients with IEI reported in the literature, the authors concluded that the majority of these were affected with primary antibody deficiency (n=446), of which 290 had underlying common variable immunodeficiency (CVID) and 71 had XLA (23). While ICU admission and mortality rates were similar in these two patient categories (15% and 8%, respectively), it is noticeable that the XLA patient group had a significantly lower median age, thereby questioning the validity of previous reports early in the pandemic suggesting a relative protection of XLA patients from the virus-induced cytokine storm characteristic of severe/critical COVID -19 (23). Collectively, these studies suggest an important protective role of B cells and antibodies in reduction of viral load, viral clearance and clinical remission in COVID-19, and underscore the susceptibility to SARS-CoV-2 in patients with IEI affecting humoral immunity.

### Vaccination of patients with XLA

Vaccine responses in patients with XLA was addressed in a small study of four patients by Hagin et al. demonstrating an expected absence of anti-S and anti-N antibodies following the Pfizer-BioNTech mRNA vaccine (24). However, an anti-S T-cell response with cytokine secretion higher than that of healthy donors, was observed following S-peptide stimulation, suggesting an intact ability to develop cellular immunity following vaccination (24). The same pattern was observed in nine patients with XLA following the mRNA-1273 (Moderna) vaccine, who showed no specific antibody response, but a robust SARS-CoV-2 specific T-cell response (25). These findings are supported by other studies of patients with IEI, including common variable immunodeficiency (CVID), who develop SARS-CoV-2-associated T cell responses in the presence of humoral immunodeficiency (26). Regrettably, the patient reported here did not receive any vaccinations against COVID-19 despite strong medical advice to do so. This probably aggravated his disease course since he would have likely developed at least some degree of protective T cell memory in response to vaccination as suggested by several studies (27, 28). Indeed, after recovery, the patient has accepted vaccination with the most recent Pfizer-BioNTech mRNA vaccine as of fall 2022 protecting against severe disease from SARS-CoV-2 subtypes BA.4 and BA.5.

## Conclusion

The outcome of SARS-CoV-2 infection in XLA spans from asymptomatic and mild disease to hospitalization, ICU admission and death. Prolonged and relapsing COVID-19 symptoms with extended viral shedding is not unusual in this group of patients. Humoral immunodeficiencies with compromised B-cell function may aggravate the outcome of COVID-19 disease, as well as prolong the duration and increase risk of viral relapse SARS-CoV-2 infection. However, no direct prognostic tool can be used as risk stratification of patients with XLA when infected by SARS-CoV-2. This demonstrates the need for focused individualized clinical assessment of SARS-CoV-2 infection in patients with XLA to improve outcome and reduce length of hospital admission. Collectively, patients with XLA may be at increased risk of severe COVID-19, particularly if immunoglobulin replacement is insufficient, and/or in the presence of chronic pulmonary changes. Both of these were present in the patient described here. The patient may have benefitted from higher IgG levels in general, although we find it unlikely that sufficient anti-SARS-CoV-2 antibody levels were present in immunoglobulin preparations during summer and fall 2021 with a production time of ~9 months from blood donation and given the relatively low level of immunity from infection and/or vaccination in the population at that time. As to the pulmonary changes and chronic obstructive lung disease in the patient, it is difficult to ascertain to what extent these factors contributed to the severe disease course. Without doubt COVID-19 vaccination would have had some protective effect due to T cell immunity and might have prevented the severe disease. Fortunately, our patient experienced a relatively good outcome of critical COVID-19 infection and has returned to his normal life and work, possibly attributed to repeated intensified antiviral and anti-inflammatory treatments against COVID-19. Given that T-cell immunity does develop following vaccination, patients with XLA should be encouraged to undergo vaccination, together with immunoglobulin replacement therapy and early treatment with available antivirals.

## Data availability statement

The original contributions presented in the study are included in the article/Supplementary Material. Further inquiries can be directed to the corresponding author.

## Ethics statement

Written informed consent was obtained from the individual(s) for the publication of any potentially identifiable images or data included in this article.

## Author contributions

TM and MD-P conceived the idea and cared for the patient, NR cared for the patient, collected clinical information and made figures, DL was responsible for clinical microbiological analyses and data, TT and TM made literature search and wrote the first draft of the manuscript with input from NR, DL and MD-P. All authors contributed to the article and approved the submitted version.

## Funding

No funding was received specifically for this study. TM is funded by the Independent Research Fund Denmark (0134-00006B), the NOVO Nordisk Foundation (NNF20OC0064890; NNF21OC0067157), and the Lundbeck Foundation (R268-2016-3927).

## Acknowledgments

We wish to thank the patient for consenting to publication of the case report. We are grateful to Dr. Henrik Kirstein Jensen, senior consultant, Department of Pulmonary Medicine, Aalborg University Hospital for the use of BAL photos. We are also grateful to Mette Mølvadgaard, senior molecular biologist, Department of Clinical Microbiology, Aalborg University Hospital, for valuable discussions and advice.

## Conflict of interest

The authors declare that the research was conducted in the absence of any commercial or financial relationships that could be construed as a potential conflict of interest.

## Publisher’s note

All claims expressed in this article are solely those of the authors and do not necessarily represent those of their affiliated organizations, or those of the publisher, the editors and the reviewers. Any product that may be evaluated in this article, or claim that may be made by its manufacturer, is not guaranteed or endorsed by the publisher.
